# Derivation of consensus inactivation status for X-linked genes from genome-wide studies

**DOI:** 10.1186/s13293-015-0053-7

**Published:** 2015-12-30

**Authors:** Bradley P. Balaton, Allison M. Cotton, Carolyn J. Brown

**Affiliations:** Department of Medical Genetics, Molecular Epigenetics Group, Life Sciences Institute, University of British Columbia, Vancouver, Canada; Department of Medical Genetics, Centre for Molecular Medicine and Therapeutics, Child and Family Research Institute, University of British Columbia, Vancouver, BC Canada

**Keywords:** X-chromosome inactivation, Dosage compensation, Escape from X-chromosome inactivation, Somatic cell hybrids, Allelic imbalance, DNA methylation

## Abstract

**Background:**

X chromosome inactivation is the epigenetic silencing of the majority of the genes on one of the X chromosomes in XX therian mammals. In humans, approximately 15 % of genes consistently escape from this inactivation and another 15 % of genes vary between individuals or tissues in whether they are subject to, or escape from, inactivation. Multiple studies have provided inactivation status calls for a large subset of the genes on the X chromosome; however, these studies vary in which genes they were able to make calls for and in some cases which call they give a specific gene.

**Methods:**

This analysis aggregated three published studies that have examined X chromosome inactivation status of genes across the X chromosome, generating consensus calls and identifying discordancies. The impact of expression level and chromosomal location on X chromosome inactivation status was also assessed.

**Results:**

Overall, we assigned a consensus XCI status 639 genes, including 78 % of protein-coding genes expressed outside of the testes, with a lower frequency for non-coding RNA and testis-specific genes. Study-specific discordancies suggest that there may be instability of XCI during cell culture and also highlight study-specific variations in call type. We observe an enrichment of discordant genes at boundaries between genes subject to and escaping from inactivation.

**Conclusions:**

This study has compiled a comprehensive list of X-chromosome inactivation statuses for genes and also discovered some biases which will help guide future studies examining X-chromosome inactivation.

**Electronic supplementary material:**

The online version of this article (doi:10.1186/s13293-015-0053-7) contains supplementary material, which is available to authorized users.

## Background

In mammals, sex is chromosomally determined with the presence or absence of the Y chromosome generally resulting in XY males and XX females. There is clear sexual dimorphism, with major contributing factors including expression of sex-linked genes and differential hormone regulation of some gene pathways [1–3]. Sex differences can have effects on disease predisposition and sensitivity to certain therapies, leading funding agencies including the NIH in the USA and Canadian Institutes of Health Research (CIHR) in Canada, to include the consideration of sex differences in their criteria for funding. The sex difference in expression of most X-linked genes is minimized by X-chromosome inactivation (XCI); however, some genes are known to escape from XCI leading to male-female expression differences, particularly in humans [4].

XCI is the inactivation of one of the two X chromosomes (X) in XX eutherian females as a form of dosage compensation between XX females and XY males [5, 6]. Which X is inactivated is randomly chosen in each cell early in development and maintained in that cell’s descendants, resulting in females being a mosaic of which parental X is inactive. XCI allows XX females and XY males to have similar levels of expression for the majority of X-linked genes [2, 7]. However, not all X-linked genes are fully inactivated on the inactive X (Xi). Different studies suggest that between 8 [8] and 15 % [9] of X-linked genes escape from XCI and are expressed from the Xi at a level at least 10 % that of the active X (Xa). Another 10 [9] to 32 % [8] of genes on the X are variable in their XCI status between individuals or tissues. Comparatively, in mice, 3–7 % of X-linked genes escape from XCI, depending on tissue and strain [10]. Such differences in which genes escape from XCI, along with other differences in XCI between mouse and human, challenge the use of mouse as a model organism for predicting the XCI status of X-linked genes in humans.

Examples of genes that escape from XCI are the genes in the pseudoautosomal region (PAR1) at the short arm terminus of the X chromosome [9]. There are two PARs on the X, and they are homologous to the PARs at the termini of the Y chromosome. These regions recombine during male meiosis and are therefore identical between the X and Y. PAR genes do not need further dosage compensation because XX females and XY males have the same copy number. Interestingly, the PAR2 genes on the long arm of the X chromosome achieve dosage equivalence differently as they are subject to XCI while also being silenced on the Y chromosome [11].

Knowing which genes escape from XCI is important because genes that escape from XCI can contribute to male-female sex differences. Multiple studies have shown an enrichment of genes with sex-biased expression on the X chromosome [2, 12, 13]. A female expression bias predominates on the X (5 % of genes); however, some X-linked genes do show a male expression bias (1.7 % of genes) [2]. Analysis of the Genotype-Tissue Expression (GTEx) pilot project data shows that most of the 29 X chromosome genes with a female bias escape from XCI, while the eight X chromosome genes showing a male expression bias were predominantly PAR located [12]. In mouse brain samples, 12 % of genes differentially expressed between the sexes are located on the sex chromosomes, and these genes have a larger fold change between males and females than other differentially expressed genes [13].

One consequence of escape from XCI and incomplete dosage compensation is that there will be altered gene expression associated with X chromosome aneuploidies. Having a single X without a Y chromosome (Turner’s syndrome) is more severe in humans than in mice [6], and this is likely linked to differences in how many genes escape from XCI between the species [4]. In patients with Klinefelter’s syndrome (XXY males), some genes that escape from XCI were found to be overexpressed and correlated with negative phenotypes [14]. Additionally, escape from XCI can affect disease susceptibility. X-linked tumor suppressor genes which escape from XCI, an example being *UTX* [15], only require one mutation to be knocked out in males but need two for females to be affected. Another example of a gene which escapes from XCI with sex-specific disease effects is *DDX3X* which has different severities of phenotype and disease mechanisms between males and females [16].

Determining which genes escape from XCI will also further our overall understanding of XCI which has been a useful model system for understanding epigenetic regulation at other loci, especially those controlled by long non-coding RNA (lncRNA). XCI is thought to be initiated by the lncRNA XIST, which is expressed specifically from the Xi. Early in development, XIST spreads along one of the X chromosomes and allows for the recruitment of histone-modifying enzymes to make cooperative silencing modifications such as H3K27me3, ubH2A, H4K20me3, and H3K9me3 (reviewed in [17]). DNA methylation (DNAm) is another epigenetic mark associated with X inactivation, and blocking DNAm with 5-azacytidine allows reactivation of X-linked genes in human-mouse hybrid cells [18]. Other lncRNAs, such as HOTAIR, are implicated in similar epigenetic regulation [19]. Understanding XIST and the epigenetic mechanisms controlling XCI may help further our understanding of how these other lncRNAs function.

The goal of this study is to integrate the results from studies that have done large-scale analyses of which genes escape from, are subject to, or variably escape from XCI and to come up with a catalog of consensus XCI status calls using the hg19 gene map. The first of the three main studies to be integrated used two methods [9]. Human-mouse hybrid cell lines with an active or inactive human X chromosome allowed the direct examination of which genes are expressed from the Xi. Comparison of the expression of each gene from the Xi cell lines to the expression from the Xa cell lines led to a call of escape from XCI when there was 10 % or more relative Xi expression. These results will be referred to as the Carrel hybrid study. The Carrel hybrid study used nine Xi hybrid cell lines and made XCI status calls for 465 genes (Table 1). Genes which escaped in only 0, 1, or 2 cell lines were called as being subject to XCI, and genes which escaped in 7, 8, or 9 cell lines were called as escaping from XCI. Genes which escaped XCI in 3 to 6 hybrid cell lines were called as variably escaping from XCI. The same publication examined the allelic ratio of X-linked expressed SNPs in fibroblast cell lines which were skewed completely for which X was inactivated, such that in a population of cells, the same allele was always on the Xa and biallelic expression would reflect escape from XCI. These results will be referred to as the Carrel SNP study [9]. The Carrel SNP study examined a panel of 40 cell lines and made XCI status calls for 84 genes, with an average of 12 informative cell lines per gene (Table 1). Genes which had less than 23 % of their cell lines escaping from XCI were called as subject to XCI while genes with over 78 % of their cell lines escaping XCI were called as escaping from XCI. Genes with between 23 and 78 % of their cell lines escaping from XCI were called as variably escaping from XCI.

**Table 1:** Sample sizes of previous studies

Study	Carrel hybrid	Carrel SNP	Cotton AI	Cotton DNAm
XCI status calls	465	84	429	406
Number of samples	9	40	99	1875
Average number of informative samples	–	12	25	–

The number of samples used and XCI status calls made per study for the Carrel hybrid, Carrel SNP, Cotton AI, and Cotton DNAm studies. The average number of informative samples was also included for the Carrel SNP and Cotton AI studies as only samples which were heterozygous at a SNP could be used for these studies

The second study looked at the expression of X-linked SNPs using microarray data to include assessment of intronic polymorphisms [8]. The allelic imbalance (AI) between the allele on the Xa and the allele on the Xi for genes which already had strong evidence for being subject to XCI was used to assess how much skewing of XCI was present in each cell line, and this was then used to calculate how much of the AI was due to mosaicism and how much was due to escape from XCI. This will be referred to as the Cotton AI study [8]. The Cotton AI study used 99 cell lines and made XCI status calls for 419 genes with an average of 25 informative samples per gene. The same thresholds were used for the AI study as the SNP study (Table 1).

The third study used CpG island methylation data from the Illumina Infinium Human Methylation450 BeadChip platform [20]. It compared the female and male DNAm levels at CpG islands at the promoters of genes known to be subject to XCI and those known to escape from XCI to develop a classifier which could predict the XCI status of other genes. This classifier was then used on genes with unknown or less evident XCI status to make new XCI status calls. This will be referred to as the Cotton DNAm study [20]. The Cotton DNAm study examined 1875 female samples and 1053 male samples, giving XCI status calls for 409 genes (and multiple transcription start sites for most genes) (Table 1). XCI status calls were given individually by tissue, and the overall XCI status call was a list of calls which were obtained in at least one tissue. An uncallable designation was used when less than 50 % of samples in that tissue had a methylation level and male-female difference within two standard deviations of the subject or escape training genes in that tissue (50 genes were left in an uncallable category because they were uncallable in over half of the tissues examined). Genes were called as subject to or escaping from XCI in a tissue if all samples that were given an XCI status call gave the same call. Genes were called as variably escaping from XCI if they had at least one sample giving each XCI status call (subject and escape). Variable escape from XCI was rare in this study with a maximum of one third of all tissues showing variable escape for any given gene.

Additional approaches to determine XCI status, which have examined fewer genes, include DNAm analysis at non-CpG sites [21], SNP expression analysis in single cells [22], RNA-FISH to detect expression from both X chromosomes [23], analysis of protein polymorphisms in clonal cells by size [24] or by enzyme activity [25], microarray analysis of cellular expression with varying numbers of X chromosomes [26], microarray analysis of expression differences between males and females [27], and allelic expression analysis of RNA-seq data from clonal cells [28].

Each of the three studies integrated in this analysis have examined over 400 different genes, and combined there is data for 639 genes. Generally, multiple studies agree, and only 47 genes show substantial discordancies between studies, which we discuss. There is an enrichment of discordancies and calls of mostly variable escape from XCI at putative XCI boundaries. Seventy percent of protein-coding messenger RNA (mRNA) genes have an XCI status call with the hypermethylated cancer-testes antigen gene family accounting for 42 % of the remaining uncalled mRNA genes. However, fewer of the non-protein-coding genes have a defined XCI status.

## Methods

### Categorization of X-linked genes

A full list of genes on the X chromosome was downloaded from University of California, Santa Cruz (UCSC)’s HG19.knownGene table browser [29]. The table was condensed manually from having an entry for each transcription start site to having an entry for each gene. XCI calls from the studies were added to the table, matching alternate gene names from the National Center for Biotechnology Information (NCBI) [30] along with using the in silico PCR tool in UCSC [31] with published primers [9].

Genes were placed into eight categories for an overall XCI status call. If all of a gene’s calls from different studies were the same, then the gene was placed in a category for all subjects, all escapes or all variable escapes. If the majority of studies (2 out of 3 or 3 out of 4) gave the same call, then the gene was placed in the mostly subject, mostly escape or mostly variable escape categories. Genes that had one-call subject or one-call escape and a variable escape call which leaned towards the same call (variable escape in a study, with less than 34 % or greater than 65 % of samples escaping XCI) were also placed in the mostly subject and mostly escape categories. The Cotton DNAm study gave some calls that were escape + variable escape or subject + variable escape; for my categorization, these genes were considered to be whichever call was given in the most tissues, this was usually subject or escape. Genes that had no calls in any of the studies were designated as the no call category, while genes that did not fit any of these other categories were placed in the discordant category. Discordant genes had either an even split of different calls or had one of each call (subject, escape, and variable escape from XCI).

Genes were sorted by their transcript type (mRNA, micro RNA (miRNA), ncRNA, snRNA, transfer ribonucleic acid (tRNA)) as determined by UCSC’s HG19.kgXref table [29] and if still unknown, a search of NCBI. A list of cancer-testis antigen genes was taken from CTdatabase [32].

To determine the source of discordancies, genes with three or four calls and only one study giving a different call from the other studies were examined. The study which gave the discordant call was noted, along with the call it gave and the call agreed upon by the other studies.

### Expression analysis

Expression data for the lymphoblast cell line GM12878 was downloaded from GEO dataset GSE30400 [28], and expression data for the fibroblast cell line IMR90 was downloaded from GEO dataset GSM981249 [33]. This data was annotated using Seqmonk (Babraham Bioinformatics) using our condensed X chromosome gene list. A Tukey test was performed to determine if expression levels in lymphoblasts differed amongst the various categories using the multcomp package in R [34, 35]. This was repeated for the calls given by each individual study.

### Domain analysis

Domains were annotated by labeling any genes between escape genes, without crossing a subject gene, as being in an escape domain and labeling any genes between subject genes without crossing an escape gene as being in a subject domain. Genes between a subject and escape gene, with no other subject or escape genes in between, were classified as boundaries; boundaries can start inside of the gene body of a gene which is subject to or escaping from XCI, as a gene’s XCI status is likely determined by its promoter. Enrichment was determined using a chi-square test (chisq.test from the MASS package in R [34, 36]). Standardized residuals were extracted from the chi-square test and used to determine enrichment of certain categories [37], followed by a chi-square test comparing the enrichment of variable, mostly variable and discordant genes in boundaries, individually against genes with no call. Genes with no call were shown to be a good control (*p* value >0.95) by a chi-square comparison between genes with no call and genes with a call, in boundaries compared to the outside of boundaries.

## Results and discussion

### Creation of a consensus XCI status

**Fig. 1:**
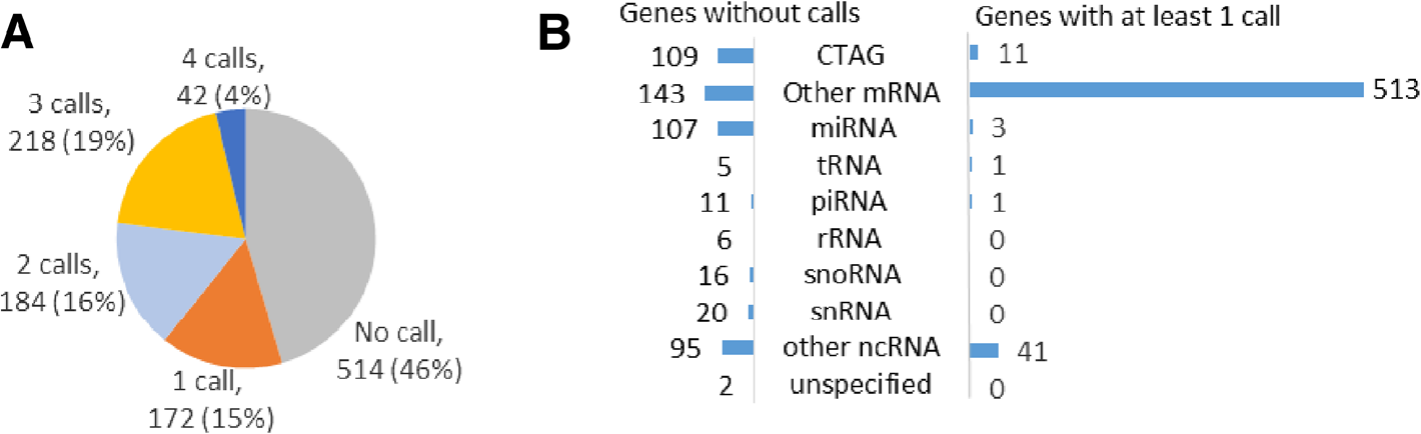
The majority of X-linked protein-coding genes have an XCI status call. **a** The number of datasets contributing an XCI status call per gene. The number of calls is the number of studies which gave an XCI status call of subject, escape, or variable escape from XCI. Genes with no call were not mentioned in any of the studies but were included in Gencode for HG19 [38, 39]. **b** The distribution of RNA transcript types for genes with and without an XCI status call. Transcript type was taken from Gencode or an NCBI search [30]. CTAG are cancer-testes antigen genes which are protein-coding genes expressed exclusively in cancer and in testes and hypermethylated in other tissues making XCI status calls very difficult. Other mRNAs are mRNA genes that are not members of the CTAG family

Gencode currently lists 1144 genes on the human X chromosome [38, 39]. Between the four datasets examined, 639 (54 %) of these genes have an XCI status call (Fig. 1a). There is a roughly equal distribution of genes that have been examined in one, two, or three of these studies; however, very few genes have an XCI status call in all four studies because the Carrel SNP study has a small sample size of 84 (Fig. 1a). Comparing the distribution of transcript types between genes with XCI status calls and those without, protein-coding genes are much more likely to have a call whereas genes for non-coding RNA such as miRNA and tRNA are more likely to not have an XCI status call (Fig. 1b). A large proportion of the protein-coding genes without a call can be explained by them belonging to the Cancer-Testis Antigen Gene (CTAG) family (Fig. 1b). CTAG genes are hypermethylated and silenced on both Xs in healthy female cells and are normally only expressed in cancer cells or in the testes of males [32]. Other genes lacking calls have very low expression (RPKM values less than 0.1) in the fibroblasts and lymphoblasts examined in the hybrid, SNP, and AI studies (102 out of 143 non-CTAG genes without a call (Additional file 1: Table S1)), and all genes without calls either are not present on or filtered out from the DNAm microarray used for assessment in the DNAm study (reasons for filtering include hypermethylation in male samples and mapping to repetitive elements or to the autosomes [20]) or were found to have methylation levels in an uncallable region between that found for known subject and escape genes. There were only 24 genes that lacked expression and were called by the DNAm but were unable to be called by the other expression studies. Enrichment of calls for protein-coding genes likely reflects the more recent identification of lncRNA genes. The smaller RNA types are too small or too tissue-specific to have their XCI status determined in these studies; furthermore, high homology to another gene might prevent assessment of XCI status and the X is enriched for large inverted repeats [40].

**Fig. 2:**
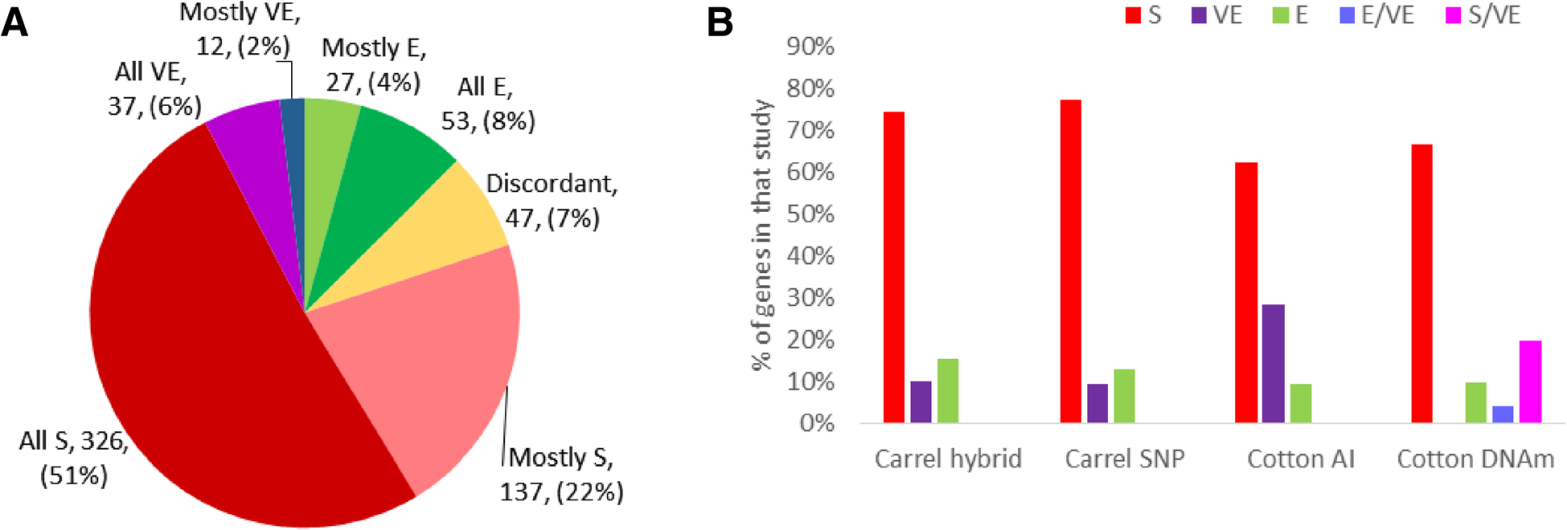
Consensus XCI status calls. **a** Distribution of our consensus XCI status calls. *E* is escape from XCI, *S* is subject to XCI, and *VE* is variably escaping from XCI in some individuals or tissues. The mostly E, S, or VE categories are genes which have two out of three or three out of four XCI status calls agree on a call of E, S, or VE and the last study disagree. The all E, S, or VE categories had at least one XCI status call for E, S, or VE and had no XCI status calls disagree. Discordant calls had either an even split of different XCI status calls or had one of each call. Genes with no call were left out of this graph. **b** The distribution of XCI status calls given by each individual study. See above for a description of E, S, and VE. E/VE and S/VE are calls from the Cotton DNAm study where most tissues were given a call of escape or subject, but some tissues were given a call of variable escape. For the sample sizes of each study see Table 1

Genes were divided into eight categories based on what XCI status the studies called the gene and how often the studies agreed (Fig. 2a). Seventy-three percent of genes were given an overall call of subject or mostly subject, roughly agreeing with the percent found to be subject in each individual study (Fig. 2b). The percent of escape and mostly escape genes (12 %) was also similar to the percent of escape genes found by each individual study. The variable escape and mostly variable escape categories (8 %) agreed with the Carrel studies; however, the Cotton studies have large differences in the amount of genes they call variable escape. This difference in the number of variable escape calls contributed to a fair amount of the discordancies between studies. Seven percent of genes on the X were discordant between studies and no consensus call could be assigned, while another 28 % had a single discordancy (categorized into one of the mostly escape, mostly subject, or mostly variable escape categories) (Fig. 2a).

### Discordancies between studies

**Fig. 3:**
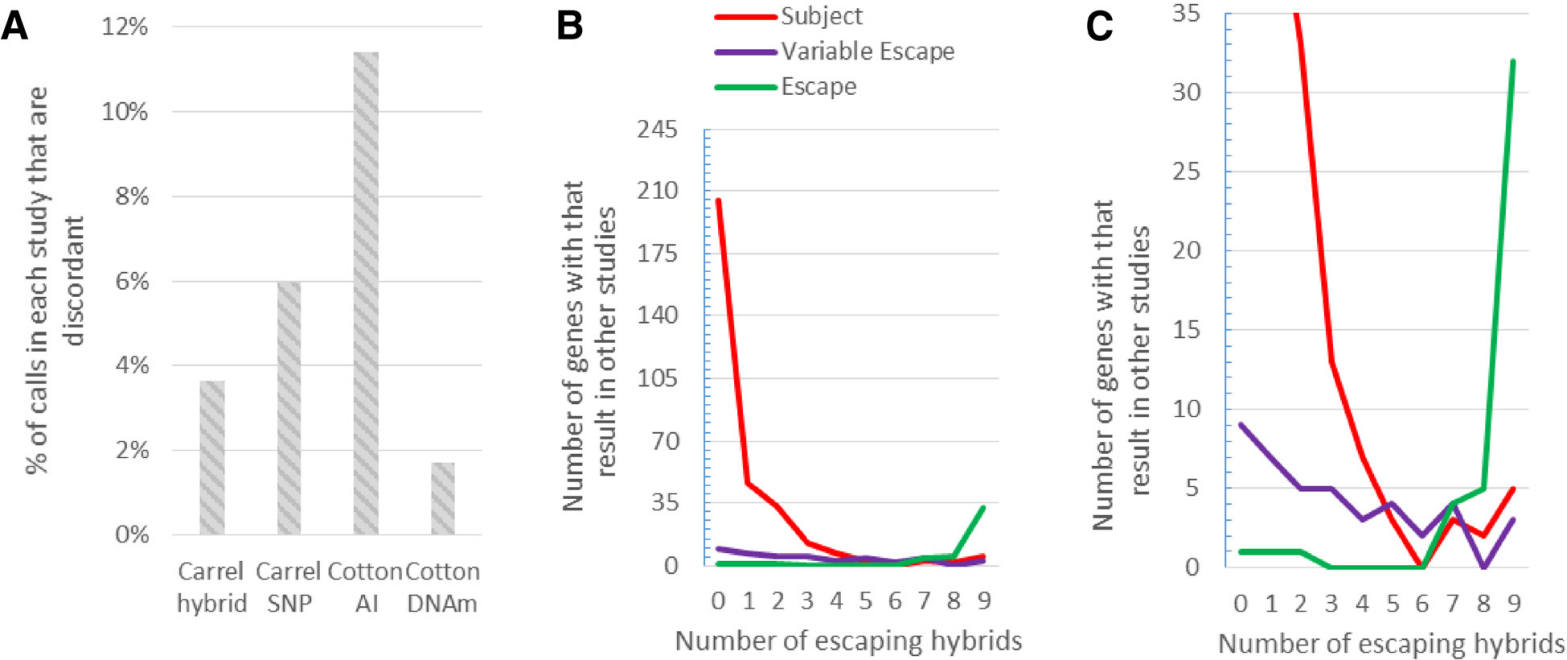
Comparison of discordancies. **a** The level of discordancies in each study. A gene is counted as discordant in a study if that study gives a call and at least two other studies agree on a different call. For the sample sizes of each study, see Table 1. **b** Comparison of the Carrel hybrid calls to calls from other studies. The number of escaping hybrids is, for each gene, in how many mouse-human hybrid cell lines (out of 9) did that gene escape XCI. The Y axis is how many genes one or more other studies agreed were subject to, escaping from, or variably escaping from XCI. **c** A magnified version of B to better show escape and variable escape from XCI

To understand the nature of the discordancies between studies, we tabulated the frequency with which studies disagreed and the difference from the consensus call (Table 2). The Cotton AI study was the most discordant study with 11 % of its calls disagreeing with two or three other studies and a tendency to call gene variable escape when other studies called that gene escape or subject (Fig. 3a). This tendency to call variable escape could be due to the extra calculations involved to correct for using cells which were only partially skewed. Another contributing factor could be that the AI study, in addition to the exonic SNPs used in the SNP study, also used intronic SNPs which are spliced out and degraded and would be present in lower levels which may affect the XCI status calls drawn from them. The AI study also used more samples than the other expression studies (an average of 25 informative samples per gene compared to 12 in the SNP study and 9 in hybrids) which would increase the chance of finding variable escape genes. The Cotton DNAm study was the most concordant study with only 2 % of its calls disagreeing with 2 or three other studies; however, it also had an uncallable category for genes which had methylation levels or male-female methylation differences between the thresholds set by training sets of known subject and escape genes (the threshold was set at two standard deviations away from the training set mean). Cotton did not give these genes a call and they were not considered in this analysis. The discordancies in the Cotton DNAm study were mostly due to it not finding any genes with a high level of variable escape from XCI (Additional file 2: Figure S1). The hybrid study discordancies arose from genes called escape or variable escape when other studies gave a subject call.

**Table 2:** Most studies show a trend with what they are calling discordantly

		Discordant Study			
Discordant call	Consensus call	Carrel hybrid	Carrel SNP	Cotton AI	Cotton DNAm
E	VE	0	1	0	2
	S	7	1	1	2
VE	E	1	1	17	0
	S	9	0	26	0
S	E	0	1	3	0
	VE	0	1	1	3

Discordant call is which XCI status call is being given by the discordant study while consensus call is the XCI status call agreed upon by two or more other studies

*E* escape from XCI, *S* subject to XCI, *VE* variable escape from XCI.

Tissue-specific differences in XCI status are an important possible source of discordancies between studies. The Carrel hybrid and SNP studies were both done in a single tissue type, fibroblasts. The Cotton AI study used both lymphoblasts and fibroblasts and found that 10 % of genes showed evidence of tissue-specific escape from XCI; these genes would not appear to be variably escaping in the Carrel studies. However, the Cotton DNAm study looked at 27 tissue types (including fibroblasts and whole blood (which includes lymphoblasts)) and found high concordancy between tissues and very few tissue-specific differences in escape from XCI. Therefore, a more likely source of differences between studies could be from differences acquired in cell culture. The Cotton DNAm study was the only study to use primary cells; the Carrel studies and Cotton AI study used cultured cells. Previous studies have shown differences in XCI between primary cells and cultured cells from the same organism [10, 41] and between individuals at different ages [42]. Genes with discordancies between studies or calls of variable escape in individual studies may be the genes most prone to epigenetic changes in culture. In the mostly subject and mostly escape categories, 90 % of the genes have variable escape as the discordant call and 82 % of the discordant genes have at least one variable escape call (Additional file 1: Table S1). This difference between the studies could also be due to differences between the methylation status and XCI status of some of the more variable genes; however, most genes which are found variable by other studies are not given an XCI status call by the Cotton DNAm study (Additional file 2: Figure S1).

The mouse-human hybrid cells may be the most different from primary cells. In hybrid cells, XIST fails to properly localize to the Xi [43]. This may reflect a loss of some heterochromatin marks on the Xi, leaving X inactivation to be maintained by fewer marks, including DNAm [44]. X-inactivated genes in hybrids are more vulnerable to reactivation by 5-azacytidine, a methylation inhibitor [18], and approximately 1 in 10^5^ hybrid cells will spontaneously reactivate the *HPRT* gene which is normally subject to inactivation [45]. Reactivation could explain the genes being called escape or variable escape in the Carrel hybrid study while being called subject in other studies. When compared with consensus calls from other studies, genes found to escape in three or four hybrid cell lines in the Carrel hybrid study (which were thus classified as variable escape in that study) are more often called subject to XCI than variably escaping from XCI (Fig. 3b, Additional file 3: Table S2). Reactivation of subject genes appears to occur for a small percentage of genes in hybrid cell lines.

Most of these studies have used expression to monitor XCI status. We therefore examined whether expression level has an effect on a gene’s XCI status call (Additional file 4: Figure S2). None of the categories had significantly different expression levels (*p* > 0.05) nor were there significant differences in expression levels for the calls in each individual study (not shown).

### Domains of escape and boundaries

**Fig. 4:**
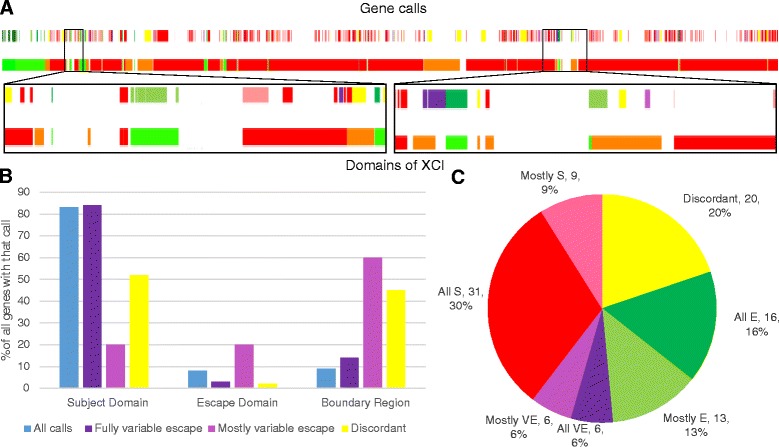
Domains of XCI and the enrichment of discordant and mostly variable escape genes at boundaries. **a** Our consensus gene calls and the domains of XCI along the X chromosome. The *top row* is the XCI status calls for all genes with a call on the X while the *second row* is the domains of XCI called from the consensus calls (see Section 2). For the XCI status calls, the colors are defined in **c**. For the domains of XCI: *red* is subject, *green* is escape, *orange* is boundaries, and *white space* is between domains. A magnification of two regions is shown below, demonstrating how genes line up with domains. Domains are defined by the first and last gene in the domain, even if they start or end inside of other genes which do not share the same domain call. See Additional files 6 and 7 for the BED files used to generate the UCSC browser track upon which this graph is based. **b** Distribution of genes into XCI status domains. The graph shows what percent of genes with each call are in each domain type. Percent is determined by dividing the number of genes with that XCI status call in that domain type by the total number of genes with that XCI status call. The all calls category includes all genes on the X chromosome, including genes with no calls. **c** Distribution of genes at boundaries. This figure includes the subject and escape genes which define the edges of the boundaries

It has been hypothesized that there are domains on the Xi with coordinately regulated XCI caused by nearby XCI way stations spreading XCI or escape elements promoting euchromatin with boundaries separating the two [46–48]. We used our categories to locate these domains and examined the domain enrichment of discordancies and variably escaping genes (Fig. 4, Additional file 5 and Additional file 6). Fully variable escape genes were most often found in subject domains at a frequency similar to the overall distribution of genes (Fig. 4b). Genes which mostly variable escape were most often in escape domains and boundary regions suggesting variation in escape genes. Discordant genes were equally abundant in subject domains and boundary regions, despite the substantially smaller size of the boundary regions. Boundaries between domains may provide clues to the mechanisms controlling XCI. Fully variable escape genes were not enriched in boundaries (*p* value >0.95) whereas mostly variable escape and discordant genes each had an approximately threefold enrichment (from 2 to 6 % of genes for mostly variable escape (*p* value <5*10^−4^) and from 7 to 20 % for discordant genes (*p* value < 4*10^−7^)) (Fig. 4c). We hypothesize that these genes may be variable due to either natural variability in the position of a boundary or from instability of boundaries due to cell culture. These discordant and variable genes are spread throughout the different boundaries; 42 % of boundaries have discordant or variable genes in them and 45 % of all the discordant genes and 60 % of all the mostly variable escape genes are in boundaries.

### Comparison to additional studies examining XCI

We compared our XCI status calls to those found by various studies examining the XCI status of single genes or regions and generally found agreement (Additional file 1: Table S1). A chi-square standardized residual analysis between the results of other studies and our analysis shows that our study was strongly enriched for calls of fully escape and mostly escape calls when other studies called a gene as escaping from XCI. Our analysis was also strongly enriched for calls of fully subject and enriched for calls of mostly subject and fully variable escape when other studies called a gene subject to XCI. When other studies disagreed with each other, our study tended to call genes discordant.

Another method of examining XCI, using non-CpG methylation (mCH), was recently reported [21] and was also compared to our results. Genes called escape by mCH were enriched for the mostly variable escape category while being strongly enriched for the escape and mostly escape categories and depleted for the subject category. Genes called subject by mCH were almost entirely in our subject and mostly subject categories. Another study used mCH to examine XCI across multiple tissue types and found tissue-specific differences [49]. Our consensus results were most concordant for genes that escaped XCI across multiple tissues. Together, these comparisons to various calls associated with XCI have shown that the XCI calls presented in our analysis are robust and are relevant to further studies.

### XCI status of genes with Y chromosome homology

The X and Y chromosomes were once a homologous pair of chromosomes, and XCI is hypothesized to provide dosage compensation as the Y homologs have decayed. The number of genes escaping XCI is higher on the evolutionarily more recent regions of the X chromosome [50], so we compared our consensus calls to which genes have been identified as having Y homologs or Y pseudogenes [51]. X-linked genes with Y homologs are enriched for genes that escape and mostly escape from XCI (Additional file 7: Figure S3A). X-linked genes with pseudogenes on the Y are not particularly enriched in any XCI category, although they have significantly less genes with no call (Additional file 7: Figure S3B). Genes with Y homologs might be anticipated to escape from XCI as having a functioning Y homolog would negate the need for dosage compensation. In addition, these genes could also have been too dosage-sensitive for the stepwise process of upregulation and becoming subject to XCI [52, reviewed in 53]. The XCI pattern for genes with Y pseudogenes may be more random, as these genes have had time to evolve XCI. Being enriched for genes with calls may be an artifact due to pseudogenes and XCI calls both being enriched for genes that are better known and well annotated.

### Our consensus XCI status calls and sex differences in expression

Genes that escape from XCI tend to not be expressed to the level that is observed from the active X chromosome. A threshold of 10 % has been used, and at this level expression from females would only be minimally higher than males; however, expression up to approximately 95 % of the Xa has been demonstrated [8], which would result in sex-biased expression. Recent genome-wide comparisons of expression across multiple tissues (GTEx [12]) tested for sex-based expression, and the results correlate well with our consensus calls. Genes with a female expression bias were strongly enriched (*p* value <10^−15^) for the escape and mostly escape from XCI categories. This makes sense as genes which escape have two transcriptionally active copies of a gene in females while only having one in males. Genes with a male expression bias are enriched for being in the PAR1 (*p* value <10^−15^) supporting the theory that there is a minor spread of inactivation into the PAR so that the Y chromosomal copy of the gene has more expression than the Xi copy [7].

## Conclusions

We have compiled a list of XCI status calls from three large studies that used different methodologies. We generated a stringent list in which multiple studies were entirely concordant for subject, escape, or variable categories. We extend those calls with a “mostly” category, allowing single discrepancies. Together, these classifications can be applied to 50 % of genes on the X, including 80 % of all non-CTAG protein-coding genes. Having a reference list of XCI statuses will prove valuable in the future as more research begins to consider sex differences and the effect of having an inactivated X chromosome. This table can be used by researchers to consider the sex effects of their genes of interest or for comparison to larger scale -omics studies such as the GTEx analysis project [12]. The table can also be informative for the impact of rearrangements, aneuploidies, or copy number variants on the Xi. This XCI status call list will also be valuable for labs such as ours studying X chromosome inactivation. Having a confident XCI status call is needed when attempting to determine patterns across genes with similar XCI statuses or when looking for boundaries between domains with differences in XCI.

## Additional files

Additional file 1: Table S1. Our consensus XCI status calls for all genes on the X chromosome. The consensus calls from this study are under the column labeled Balaton consensus calls. The data used for the rest of the analyses in this article are also included as columns. The second sheet has descriptions of each column. (XLSX 316 kb) Additional file 2: Figure S1. Comparing the Cotton DNAm XCI status calls and consensus calls. No data reflects genes which were not called in the DNAm study, primarily due to a lack of CpG islands. Uncallable are genes which had methylation between the subject and escape classifiers and were unable to be confidently called by the DNAm study. S, E, and VE are subject, escape, and variable escape from XCI. E/VE and S/VE are genes which were fully subject or escape in some tissues while variably escaping in other tissues. All 4 states were genes which had some tissues subject, escaping, variably escaping and uncallable making the gene not fit into any other XCI status category. A) The Cotton DNAm XCI status calls when the consensus call is variable escape or discordant. N = 91. B) The Cotton DNAm XCI status calls for all genes on the X chromosome for comparison. N = 1144. (PDF 126 kb) Additional file 3: Table S2. The hybrid study tends to call genes variable escape discordantly. The data used to create Fig. 4. Escaping hybrids is how many human-mouse hybrid cell lines (out of 9) were found to escape from XCI by Carrel, Hybrid call is the XCI status call from the Carrel hybrid study, % agreement is the percent of genes with that number of escaping hybrids whose Carrel hybrid call agrees with one or more other study’s call. Consensus S, VE, and E are how many genes have other studies agree on a call of subject, variable escape or escape. (DOC 37 kb) Additional file 4: Figure S2. Expression in GM12878 does not correlate with consensus XCI status call. A box and whisker plot of the log reads per kilobase of transcript per million mapped reads (RPKM) of expression. A value of 1 RPKM was added to each gene in order to include genes with 0 expression in a graph of log_10_(RPKM). E, VE, S and PAR are escape, variable escape, and subject to XCI and pseudoautosomal region. The N are: Discordant = 44, E = 29, mostly E = 26, mostly S = 129, mostly VE = 10, no call = 509, PAR = 22, S = 331, VE = 37. (PDF 89 kb) Additional file 5:
**BED file used to make a UCSC browser track with color coded consensus XCI status calls for each gene on the X (excluding genes with no XCI status calls).** This file was used to generate Fig. 4a and colors correspond to Fig. 4a. (BED 39 kb) Additional file 6:
**BED file used to make a UCSC browser track of the XCI domains.** This file was used to generate Fig. 4a and colors correspond to Fig. 4a. (BED 5 kb) Additional file 7: Figure S3. Consensus XCI status calls of genes with Y homologs or Y pseudogenes. A) XCI status calls of X genes with homologs on the Y chromosome. E is genes which escape from XCI in all studies, mostly E is genes which escape from XCI in the majority of studies, S is genes which are subject to XCI in all studies, discordant is genes which either have an even split of S and E calls or have one of each call (including variable escape), and no call is genes with no XCI status call in any study. N = 19. B) XCI status calls of X genes with pseudogenes on the Y chromosome. See above for description of most categories. VE and mostly VE is variable escape from XCI in all studies and variable escape from XCI in the majority of studies. Mostly S is subject to XCI in the majority of studies. N = 264. (PDF 106 kb)

## Abbreviations

AI: allelic imbalance
CTAG: cancer-testes antigen gene
DNAm: DNA methylation
GTEx: Genotype-Tissue Expression
lncRNA: long non-coding RNA
mCH: non-CpG methylation
PAR: pseudoautosomal region
X: X chromosome
Xa: active X chromosome
XCI: X chromosome inactivation
Xi: inactive X chromosome
