# Supplementary figures and images for: Derivation of consensus inactivation status for X-linked genes from genome-wide studies
Source: Biol Sex Differ. 2015 Dec 30;6:35. doi: 10.1186/s13293-015-0053-7 (PMC4696107; doi:10.1186/s13293-015-0053-7)

A)

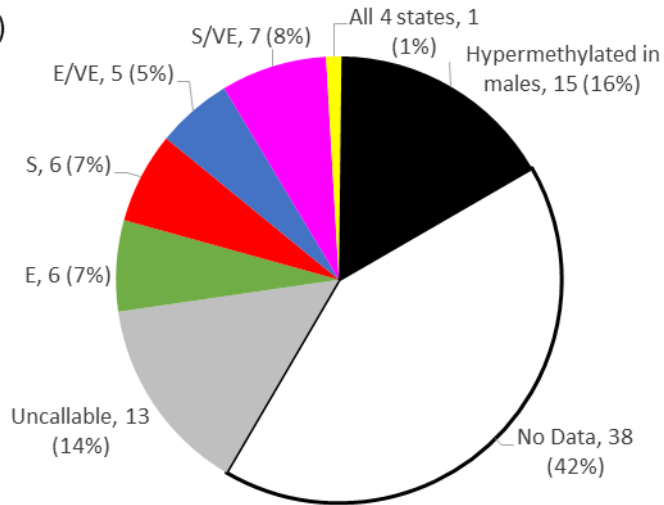

B)

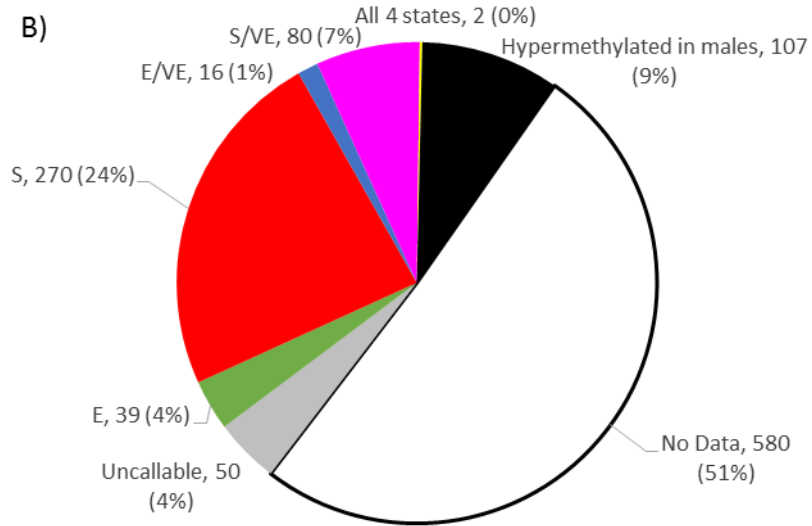

Supplement: Additional file 2: Figure S1. — Comparing the Cotton DNAm XCI status calls and consensus calls. No data reflects genes which were not called in the DNAm study, primarily due to a lack of CpG islands. Uncallable are genes which had methylation between the subject and escape classifiers and were unable to be confidently called by the DNAm study. S, E, and VE are subject, escape, and variable escape from XCI. E/VE and S/VE are genes which were fully subject or escape in some tissues while variably escaping in other tissues. All 4 states were genes which had some tissues subject, escaping, variably escaping and uncallable making the gene not fit into any other XCI status category. A) The Cotton DNAm XCI status calls when the consensus call is variable escape or discordant. N = 91. B) The Cotton DNAm XCI status calls for all genes on the X chromosome for comparison. N = 1144. (PDF 126 kb) [file 13293_2015_53_MOESM2_ESM.pdf]

Expression vs XCI status

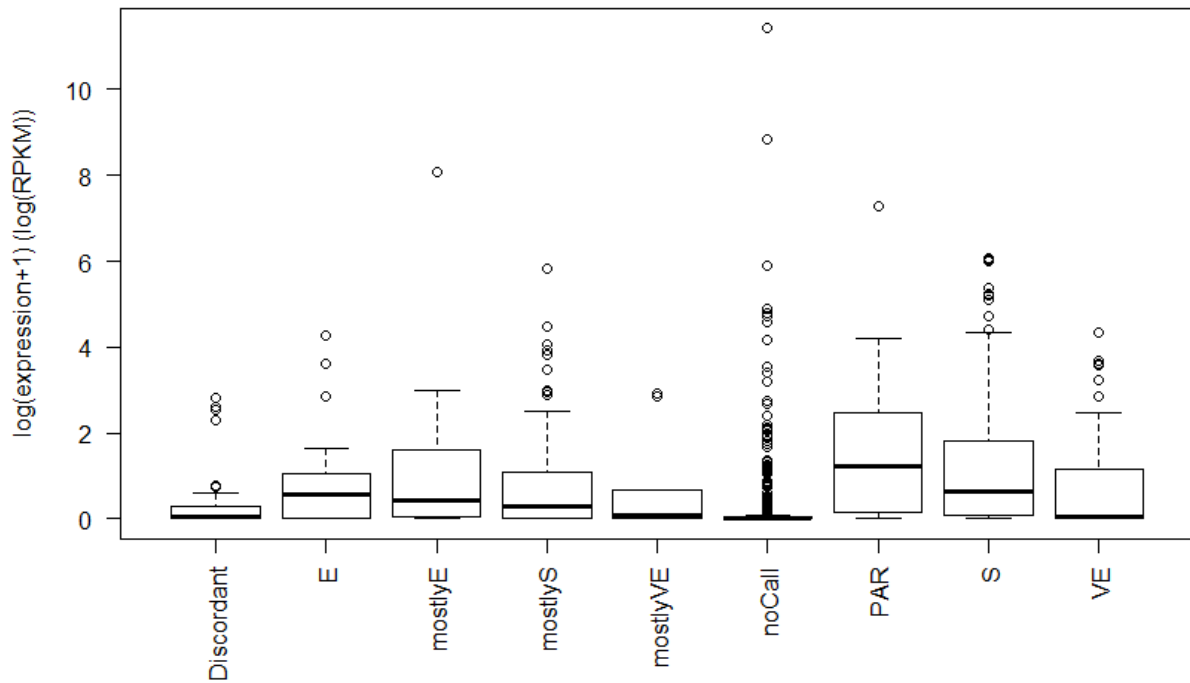

Supplement: Additional file 4: Figure S2. — Expression in GM12878 does not correlate with consensus XCI status call. A box and whisker plot of the log reads per kilobase of transcript per million mapped reads (RPKM) of expression. A value of 1 RPKM was added to each gene in order to include genes with 0 expression in a graph of log10(RPKM). E, VE, S and PAR are escape, variable escape, and subject to XCI and pseudoautosomal region. The N are: Discordant = 44, E = 29, mostly E = 26, mostly S = 129, mostly VE = 10, no call = 509, PAR = 22, S = 331, VE = 37. (PDF 89 kb) [file 13293_2015_53_MOESM4_ESM.pdf]

A)

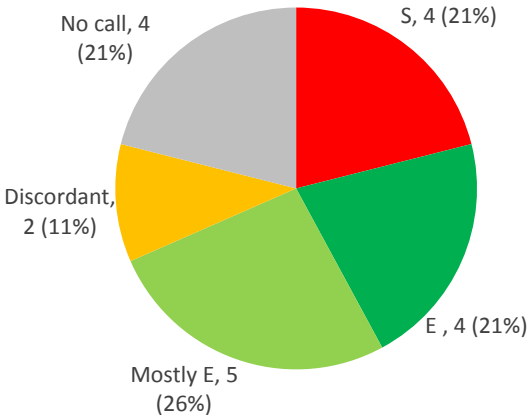

B)

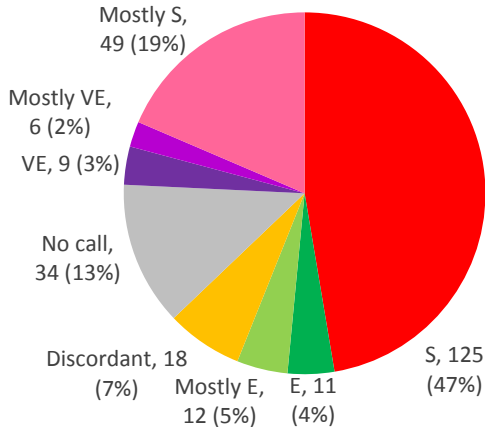

Supplement: Additional file 7: Figure S3. — Consensus XCI status calls of genes with Y homologs or Y pseudogenes. A) XCI status calls of X genes with homologs on the Y chromosome. E is genes which escape from XCI in all studies, mostly E is genes which escape from XCI in the majority of studies, S is genes which are subject to XCI in all studies, discordant is genes which either have an even split of S and E calls or have one of each call (including variable escape), and no call is genes with no XCI status call in any study. N = 19. B) XCI status calls of X genes with pseudogenes on the Y chromosome. See above for description of most categories. VE and mostly VE is variable escape from XCI in all studies and variable escape from XCI in the majority of studies. Mostly S is subject to XCI in the majority of studies. N = 264. (PDF 106 kb) [file 13293_2015_53_MOESM7_ESM.pdf]
