# Supplementary material for: Derivation of consensus inactivation status for X-linked genes from genome-wide studies
Source: Biol Sex Differ. 2015 Dec 30;6:35. doi: 10.1186/s13293-015-0053-7 (PMC4696107; doi:10.1186/s13293-015-0053-7)
Supplement: Additional file 3: Table S2. — The hybrid study tends to call genes variable escape discordantly. The data used to create Fig. 4. Escaping hybrids is how many human-mouse hybrid cell lines (out of 9) were found to escape from XCI by Carrel, Hybrid call is the XCI status call from the Carrel hybrid study, % agreement is the percent of genes with that number of escaping hybrids whose Carrel hybrid call agrees with one or more other study’s call. Consensus S, VE, and E are how many genes have other studies agree on a call of subject, variable escape or escape. (DOC 37 kb) [file 13293_2015_53_MOESM3_ESM.doc]

| Escaping hybrids | Hybrid call | % agreement | Consensus S | Consensus VE | Consensus E |
| --- | --- | --- | --- | --- | --- |
| 0 | S | 95 | 205 | 9 | 1 |
| 1 | S | 85 | 46 | 7 | 1 |
| 2 | S | 85 | 33 | 5 | 1 |
| 3 | Ve | 28 | 13 | 5 | 0 |
| 4 | Ve | 30 | 7 | 3 | 0 |
| 5 | Ve | 57 | 3 | 4 | 0 |
| 6 | Ve | 100 | 0 | 2 | 0 |
| 7 | E | 36 | 3 | 4 | 4 |
| 8 | E | 71 | 2 | 0 | 5 |
| 9 | E | 80 | 5 | 3 | 32 |
